# Building Bridges between Pharmacy and Psychosocial Care: Supporting and Referring Patients with Psychosocial Needs in a Pilot Study with Community Pharmacists

**DOI:** 10.5334/ijic.7531

**Published:** 2023-09-25

**Authors:** Eva Rens, Janne Scheepers, Veerle Foulon, Caroline Hutsebaut, Aline Ghijselings, Kris Van den Broeck

**Affiliations:** 1 Family and Population Health (FAMPOP), University of Antwerp, Belgium; 2 Department of Pharmaceutical and Pharmacological Sciences, KU Leuven, Belgium; 3 Vlaams Apothekersnetwerk (Flemish Association of Pharmacists), Belgium

**Keywords:** pharmacy, social work, unmet need, mental health, psychosocial, integrated care

## Abstract

**Introduction:**

Community pharmacists are accessible primary care providers and therefore in a good position to detect unmet psychosocial needs of their patients and pharmacy visitors.

**Description:**

A collaboration between pharmacists and psychosocial work was set up in Flanders, Belgium. Community pharmacists were trained to discuss psychosocial needs, to inform patients about possible help and refer them to a Center for General Wellbeing if needed. During the pilot of the project between October 2021 and January 2022, the feasibility and potential of this collaboration were examined.

**Discussion:**

A total of 79 patient contacts about psychosocial wellbeing were reported using an online registration form, the majority of which concerned women. Family problems and mental health problems were most often reported. Focus group discussions with 28 participating pharmacists showed that they experience their role in psychosocial care as fulfilling and of valuable. Patient satisfaction was mentioned to be a major motivating factor, while time and privacy are barriers. Adequate training in psychosocial wellbeing and care was considered crucial.

**Conclusion:**

Pharmacists can be valuable partners in the recognition and referral of patients with unmet psychosocial needs. Structural collaborations between community pharmacy and psychosocial care should be further supported.

## Introduction

The original medication-centered basis of the pharmacist has evolved to a patient-centered one. Pharmacists are increasingly depended on for healthcare advice, counseling and direct care [1,2]. Simultaneously, there is a growing awareness that community pharmacists can play a role in the wellbeing of the population beyond medication management [3,4]. Given that community pharmacists are highly accessible healthcare professionals who come in contact with a large number of vulnerable people on a daily basis, they are in an ideal position to detect unmet psychosocial needs of their patients. Psychosocial needs include the broad spectrum of all complaints that are not strictly medical, and encompass any combination of mental health, social-economical, emotional or behavioral needs. They affect the patient’s functioning in daily life, his or her environment and/or life events [5].

Psychosocial needs often remain undetected and unmet. Some factors that can account for this ‘care gap’ include stigma, low motivation, not knowing where to seek help or a lack of insight into one’s own needs. For example, it is known that only half of people with a need for mental health care seek formal care, resulting in a prevalence of approximately 5% unmet needs for mental health care in the general population [6,7]. Psychosocial problems may interfere with adequate treatment of somatic problems and those in a social-economically disadvantaged position are generally more prone to have their health needs unmet [8]. Moreover, patients with unmet social needs, such as financial strain or a lack of personal safety, have decreased perceived ability to manage general health problems [9].

Pharmacists are nowadays incorporated in primary health care teams and networks, and can therefore closely and locally cooperate with community mental health and social care settings [10]. However, there is considerable variation in the current practice and the support that pharmacists offer to patients with mental health needs, ranging from solely dispensing medication to providing broader primary mental health support and signposting and referrals to other health providers. A qualitative study showed that this is highly dependent on their overall perception of their role in primary mental health care and what they consider to be patients’ needs [11].

Although little is known about the role of pharmacists in promoting psychosocial wellbeing, a review dating from 2003 showed that community pharmacy can successfully cooperate with mental health care, leading to substantial improvements in patient care and wellbeing [12]. For example, community pharmacy services significantly improved the patients’ medication adherence and treatment satisfaction, their mental health-related quality of life and their overall perception of illness [13,14]. Pharmacists have also successfully demonstrated that they can add value to the early detection and referral of individuals with depression through screening [15]. Also during the COVID-19 pandemic, codewords were even launched in some European countries such that domestic abuse victims visiting a pharmacist could discreetly ask for help.

However, despite the high potential of pharmacists in psychosocial care, qualitative research with community pharmacists has shown that they feel that they are not yet fully realizing this potential regarding the care for people with mental health problems or addictions, especially due to limitations within the work environment and lack of structural implementations [16]. A systematic review of mental health training programs for community pharmacists and pharmacy students highlighted the importance of adequate training and found that this not only led to the improvement in pharmacists’ knowledge, but also to a reduction in their stigmatic beliefs and attitudes [17]. Another recent qualitative exploration with community pharmacists investigated the delivery of mental health services in pharmacies, and found that collaboration with other primary care providers as well as training and education in mental health are major facilitators [10]. Identified barriers included a lack of support, time, privacy and remuneration.

A Belgian survey study showed that although pharmacists generally have a positive attitude towards depression care, they report providing less care to people with depression compared to those with other illnesses. Barriers included a lack of information about the person and their treatment, the lack of education on this topic, and the lack of time and privacy in the pharmacy [18]. A lack of training in mental health issues was the most important barrier reported in another Belgian survey study, leading to a low level of cooperation with general practitioners in depression care [19]. Finally, a survey study with pharmacists in the US showed that four in ten indicate that the emphasis on mental health in their pharmacist training may have been inadequate [20].

To further explore the role of the pharmacist in the detection and referral of unmet psychosocial needs, the Flemish Government funded a project in Flanders (Belgium), named ‘#CAVAsa’, in which a collaboration was set up between community pharmacy and a social work organization. This is the first project in which pharmacists are actively involved in psychosocial care, especially as projects with pharmacists that are not directly related to medication are rare. In line with this, a qualitative study with Belgian primary care providers concluded that multidisciplinary collaboration and assistance to patients with psychosocial problems falls short [5].

Community pharmacists were trained to detect psychosocial needs, and to inform and help patients. If needed, they could refer their patients to a local Flemish Center for General Wellbeing called “CAW”. The CAWs provide professional advice and primary psychosocial care for all problems relating to general wellbeing. Both short-term counseling and long-term guidance are possible. The patient’s informal network is involved as well when this is accepted by the patient. Furthermore, the CAWs can also provide shelter in situations of homelessness or domestic violence, and they have an extensive professional network including more specialized mental health care, judicial and financial support services, children and youth care and family care. If secondary care is indicated, the CAWs explore the possible care pathways in close collaboration with the patient.

In this paper, we report on how the pharmacists experienced the project. Also, it describes the number of patient contacts about psychosocial wellbeing, the types of psychosocial problems pharmacists encountered the most, and the duration and subjective evaluation of the conversations.

## Description of care practice

Participation in the project was possible for all pharmacists currently working in a community pharmacy in one of nine Flemish primary care zones (i.e., regions of approximately 125.000 citizens in which primary care providers work closely together) spread over each province in Flanders. Recruitment of pharmacists was done through the coordinators of the primary care zones and through personal recruitment (phone calls) by the project team. A total of 71 pharmacists agreed to participate and no pharmacists were excluded.

Before the start of the study period, the participating pharmacists received a mandatory training consisting of two two-hour online webinars in which an overview of the prevalence, risk factors and signs of various psychosocial problems was given. Conversation techniques and the referral method of CAW were discussed using patient case studies. All pharmacists followed this training, either by livestream or by watching the recordings in the month after the webinars. To further support pharmacists in their role, an awareness-raising poster, informative patient leaflet and online registration system were developed.

From October 1^st^ 2021 until January 28^th^ 2022, pharmacists were asked to be alert for signs of psychosocial problems in their pharmacy visitors and to open the conversation about wellbeing when they felt someone might have a need for psychosocial help. The detection of needs was based mainly on spontaneous conversations with the patient, but also by paying extra attention to alarming signs (e.g., payment problems or poor hygiene) and increased vigilance in case of a negative gut feeling. The eligible psychosocial problems of patients are very broad. In general, there is a focus on patients with socioeconomic and mental health challenges who would benefit from primary psychosocial health care. Psychosocial problems captured by the project include mental health problems, substance abuse, financial problems, family problems, and help for victims and offenders of domestic violence.

When a possible psychosocial need was perceived, the pharmacist carefully addressed this by expressing concern and asking probing questions. When pharmacists believed that help was needed, they informed the patient about the project and the services of the CAW and a leaflet was discreetly handed over with more information about the project and care provided by the CAWs. If the patient was interested, the pharmacist arranged a referral to a local CAW, after which the social workers of the CAW contacted the patient. Referrals were done via the online page for professionals on the CAW website. Explicit verbal permission was always requested for referrals.

### Evaluation

Pharmacists registered all patient encounters in the context of the project by filling in an online Qualtrics (Provo, UT, https://www.qualtrics.com) form. This registration was used solely for research purposes and not for referrals. No patient-identifying information was collected, so the patient remained anonymous for the researchers and therefore no patient consent was required for this purpose. All pharmacists who registered at least one patient contact in the online form (i.e., not necessarily a direct referral) received a remuneration of €150 per pharmacy practice for their engagement. The following variables were recorded:

Estimated conversation duration in minutes

- Patient gender (M/F)
- Patient age category
- Presumed psychosocial patient needs (multiple answers possible)
  - Financial problems
  - Family problems (without suspicion of domestic abuse)
  - Family problems (with suspicion of domestic abuse)
  - Mental health problems
  - Alcohol or substance abuse
  - Unclear
  - Other (specify)
- What did the intervention consist of (multiple answers possible)
  - Listening to the patient
  - Handing over a leaflet
  - Informing about CAW
  - Referral to CAW via the CAW website
  - Other (specify)
- Where did the conversation take place? (at the pharmacy counter/separate room)
- Was this the first contact with the patient in the context of the project? (yes/no)
- How did the conversation go? (very good/rather good/rather difficult/very difficult)

Moreover, qualitative research was conducted to explore the pharmacists’ perceptions of the project. During the second month of the pilot study phase, all participating pharmacists were invited to focus group discussions through e-mail to share how the project was going in their practice and how they experienced it. Seven online focus group discussions were organized with a total of 28 pharmacists, 9 CAW employees and two organizational members of the primary care zones. The semi-structured discussion guide included the following themes to explore the pharmacists’ perceptions of the project:

- How does the pharmacist team experience the tools provided for the project?
- How does the pharmacist team experience the detection of psychosocial problems?
- How does the pharmacist team experience the implementation of the intervention?
- How does the pharmacist team view the expansion of project #CAVAsa to more pharmacies in Flanders?
- To what extent has the training removed the barriers for pharmacists to go into dialogue with citizens with (unmet) psychosocial needs?

Participants were encouraged to share their experiences. Internal barriers such as communication with the project team and the use of the developed materials were discussed as well.

The focus group discussions were led by the Master student JS who was supported by a senior team member experienced in qualitative research. The discussions were processed ad verbatim, in addition to notes taken by the project team, and thematically analyzed using the principles of framework analysis [21]. For this purpose, the data were read through several times and then coded with the software package NVivo® (version 1.6.1) by the master student pharmacy (JS), whereby a codebook and code tree were created during the process (inductive coding). It was found that no new information emerged after seven focus groups, suggesting data saturation. The multidisciplinary project team, consisting of a pharmacist/scientific assistant (CH), a master student pharmacy (JS), a psychologist/scientific assistant (ER) and an engineer/project coordinator (AG), interpreted the findings both individually and in dialogue. Upon completion of all focus groups, a brief summary of the findings was sent to all pharmacists and no additional feedback was received. The qualitative research was approved as a master’s thesis (MP018081) by the Research Ethics Committee UZ/KU Leuven.

### Results

Of the 71 pharmacists who agreed to participate in the project and received the training, six pharmacists withdrew during the project, mainly due to time constraints. No demographic information was collected about the pharmacists.

#### Registrations

A total of 79 patient contacts related to psychosocial wellbeing were registered during the four month study phase. The majority of these patients were women (73.4%, n = 51). Almost half of the patients were between 46 and 64 years old (44.3%, n = 35), 20 patients were between 65 and 85 years old, 19 patients were between 26 and 45 years old, four patients were younger than 26 years old and only one patient was older than 85.

**Figure 1:**
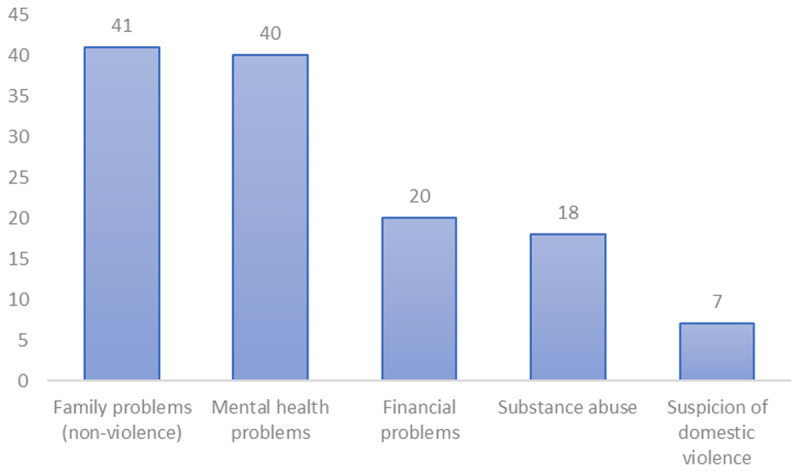
Number of registrations per type of psychosocial need (N = 79).

The number of patient contacts per psychosocial need type is shown in Figure 1. Pharmacists could indicate multiple needs per patient contact, and a total 126 needs were reported across all patients. In respectively 41 and 40 out of the total of 79 patient contacts about psychosocial wellbeing, family problems (without a suspicion of domestic violence) and/or mental health problems were mentioned. Financial problems and substance abuse were reported 20 and 18 times, respectively. Domestic violence was suspected in seven cases. Only one pharmacists found it unclear to indicate a specific psychosocial need. Moreover, ‘Other’ (e.g., loss of a family member, socially isolated, housing problem) was chosen six times.

In 65 of the 79 registered patient contacts, an information leaflet was handed to the patient. Information about the CAW was given 53 times. Ten patients were directly referred to a CAW. No data were collected on the follow-up of care after referral.

Pharmacists had to estimate the conversation duration by selecting a category. The conversation generally lasted between 5 and 15 minutes, with a median estimated duration of 11 – 15 minutes. The vast majority of the conversations (87.3%, n = 69) took place at the pharmacy counter instead of in a separate room. Almost all conversations were first conversations in the context of the project, except for two follow-up conversations. As regards the subjective evaluation of the conversation, the pharmacist indicated that the conversation went ‘rather good’ or ‘very good’ in 86% of the registered patient contacts. Ten contacts were described as ‘rather difficult’, and one as ‘very difficult’.

#### Focus group discussions

A total of 28 pharmacists participated in the focus group discussions. This accounts for 42% of all pharmacists in the project. The limited attendance might be due to the busy covid-related circumstances. All focus groups lasted between 90 and 120 minutes. Four themes were identified after the framework analysis:

1. the role of pharmacists in psychosocial wellbeing
2. the detection of psychosocial needs in the pharmacy
3. barriers to implementing psychosocial care in the pharmacists’ role
4. motivators in implementing psychosocial care in the pharmacists’ role

##### The role of pharmacists in psychosocial wellbeing

The pharmacists stressed that counselling for psychosocial problems does not fall within their area of expertise, but that a role in the detection and referral of these problems is valuable. Some pharmacists saw this role as an extension of their regular scope of practice, whereas others argued that helping psychosocially vulnerable patients was already part of their job. Before the start of the project, they often felt unsure about dealing with psychosocial problems. Pharmacists found it helpful that, because of the project, they had a better knowledge of psychosocial wellbeing and that they could refer patients directly to appropriate care. Another added value of this role is that it also raises awareness among the general public of the pharmacist as a full-fledged healthcare- and wellbeing professional.

*“I would then say, ‘I have a concern, something we would like to help you with, but that’s not my expertise. My specialty is giving health advice, medication guidance. I can do a lot of listening but we need to leave this to people who are really versed in this and are specialists. They’re going to be able to help you along much better.’” (FGD2, woman)*

*“That is also a bit the intention of that project, I think. That is the idea behind putting up this poster, to make our role known, our role of listening and also of referring people.” (FGD1, man)*

##### The detection and referral of psychosocial needs in the pharmacy

In terms of detecting psychosocially vulnerable patients, most pharmacists felt confident. However, starting a conversation about the detected needs seemed more difficult and many indicated that they would only address the patient’s problems if the patient asked for help. However, pharmacists noticed that patients generally appreciate it when a sensitive conversation is initiated, just because patients do not always dare to bring it up themselves. Pharmacists found it easier to start a conversation with well-known patients than with casual visitors.

*“It’s more when the patient starts to tell something or discloses something, that we follow up on that. And I think that also has to do with the fact that there is no full privacy. […] I find it very difficult to start talking about that myself, while the other person is not disclosing anything.” (FGD1, woman)*

*“Detecting that something is not right, that’s not difficult, but it’s then starting the conversation that is time-consuming. All your questions actually, you also have to be able to do that a bit discreetly.” (FGD3, woman)*

##### Barriers to implementing psychosocial care in the pharmacists’ role

Lack of time is a major barrier. The hectic study period, during the COVID-19 pandemic and the flu season, played a particularly important role. As a result, the focus on psychosocial well-being sometimes shifted to the background and interventions were not always registered on the online platform. Another barrier is that a pharmacy is a public space where full privacy is not guaranteed. If there are other patients in the pharmacy or in the queue, it is difficult to have a lengthy or sensitive conversation. A solution is to discuss this in a separate room, but this is not always possible due to staff shortage. In addition, some pharmacists fear that they are crossing a line when addressing psychosocial problems, or feel that suggesting professional help may be experienced as stigmatizing the patient. Domestic violence and financial problems were particularly difficult topics.

*“I am alone in the pharmacy and I think there is less time to listen to patients, especially when there are three to four people behind in the line.” (FGD6, woman)*

*“I find domestic violence the most difficult, because that remains the most hidden. People keep quiet about it the most, so to detect it is the most difficult thing for me.” (FGD2, woman)*

##### Motivators in implementing psychosocial care in the pharmacists’ role

Perceived patient satisfaction and successful conversations were major motivations. Knowing that the referred patient was helped well was an important motivation. Pharmacists mentioned that they felt that some patients were grateful for their help, and that this provided extra job fulfillment. The possibility to arrange more targeted referrals is a major plus. Pharmacists agreed that it is helpful to meet personally with staff from the local CAW beforehand. The training and materials developed for the project, particularly the information leaflet for patients, facilitated the role as well. The majority felt that a financial incentive was unnecessary, although some thought that this could be an extra motivation.

*“Before [the start of the project], we could call the general practitioner or something, but that’s really where it stopped. That’s why I was actually very happy to be able to broaden that range of tasks, so that when you have someone in front of you with a psychosocial need, you don’t have to feel bad about “that person has left now, but I haven’t actually been able to do much.’” (FGD5, woman)*

*“When you then try to start that conversation, people – I’m not saying everyone – but that person was then grateful that someone was thinking along with him, like ‘is there anything that could possibly be done about it?” (FGD2, woman)*

## Discussion

This pilot study examined the potential of a collaboration between community pharmacy and psychosocial care. Community pharmacists were trained to detect patients with possible unmet psychosocial needs, to inform them about possible help and refer them to a Center for General Welfare Work (Flemish CAW) if needed. Moreover, this aligns with the Flemish aim to install more community-oriented integrated care and to improve access to care for vulnerable and hard-to-reach groups.

Note that the majority of studies on this topic in the literature are largely about the role of pharmacists in primary mental health care specifically, while the project discussed in this paper also aims to reach the broader group of individuals with psychological and social problems and vulnerabilities. The target patient group was deliberately kept broad to help as many patients as possible with unmet needs, with special attention to vulnerable people. This decision was also made based on the idea that wellbeing encompasses psychological as well as environmental factors (e.g., family factors) and enabling factors (e.g., financial factors).

Some important contextual remarks should be made when interpreting the findings. First, the study period coincided with a wave of COVID-19 and the flu season. Pharmacists were deployed to provide information about and perform antigen testing and were actively involved in a new vaccination strategy for COVID-19. The participating pharmacists experienced an exceptionally busy and hectic period. As a result, pharmacists admitted that the project was often inevitably shifted to the background. This may have led to both an under-detection and under-reporting of psychosocial problems, because pharmacists did not always have the time to start a conversation about psychosocial wellbeing or to register patient contacts about psychosocial problems on the study platform.

It was found that pharmacists especially discuss psychosocial wellbeing with female pharmacy visitors. It is unclear why this is the case and we cannot use our data to conclude that psychosocial needs are higher among women as compared to men, as our research was explorative and many other reasons can explain this finding (e.g., the possibility that more women than men visit the pharmacy). For example, women tend to be more open about their emotions, and might therefore be more willing to start a conversation about their personal problems [22]. Also pharmacist-level factors may account for this difference, for example that pharmacists perceive women as more vulnerable or more prone to have an unmet need. These possibilities require more rigorous investigation in further research.

Mental health problems and family problems without a suspicion of violence were most often reported in the registrations of patient contacts. This seems consistent with prevalence estimates of various psychosocial issues, but again, other factors can be at play, such as that these problems are easier to recognize and discuss. Moreover, given that approximately one in ten Flemish people has financial problems, one in eight experiences domestic violence (including verbal or emotional violence), one in seven has a mental disorder, and one in seven has problematic alcohol use, more than 79 registrations could have been expected in a four-month period [23].

This discrepancy can, besides time constraints, possibly be explained by the fact that pharmacists primarily engage with patients who initiate the conversation about psychosocial wellbeing. It can be assumed that a minority of patients discloses psychosocial problems spontaneously themselves, and an even smaller proportion will directly request the pharmacist for help in these cases. Although the pharmacists generally feel confident in detecting psychosocial vulnerability, bringing up the topic themselves was considered more difficult. There seems to be a tension between wanting to help the patient and the fear that the patient might be displeased with the pharmacists’ questions about wellbeing.

Ten patients were formally referred to a CAW. The rather low referral rate possibly shows that it may be difficult to convince patients to get professional help in one conversation. On the other hand, during the conversation it may turn out that professional help is not needed or that the patient is already getting professional help. In the focus groups, some pharmacists indeed mentioned that a direct referral was not considered necessary or that handing over a leaflet was thought to be sufficient. An earlier qualitative study with primary care professionals in Flanders concluded that referrals are often complicated by stigma and practical constraints [5].

Some pharmacists see their role in detecting and referring patients with a psychosocial need as an inherent part of their job as primary health care providers, while others see it more as an extension of their duties. This was also found in another qualitative study with community pharmacists, which showed that pharmacists’ beliefs about providing mental health services vary strongly, with some pharmacists arguing that a role in mental health care beyond the dispensing of medicines pushes the boundaries of their role [10].

The participating pharmacists agree that paying particular attention to psychosocial wellbeing of their patients provides great value, with patient satisfaction being the main motivation. This is confirmed by earlier research, in which it was found that Belgian pharmacists endorse a positive attitude toward their potential role in depression care [18,19]. A narrative review investigating the new role of the pharmacist in community mental health indicated that highlighting the role of the pharmacist as a confidential care provider was considered the primary facilitator for the implementation of mental health services in pharmacy according to pharmacists [3]. A similar motivation was mentioned in the focus groups: pharmacists thought it is positive that this project emphasizes the role of the pharmacist as a well-being actor to the population. This was found in an Australian qualitative study about mental health services in pharmacies as well, where community pharmacists highlighted the need to promote their role in mental health and to increase community awareness [9]. However, the role of the pharmacist should also be adequately delineated: psychosocial care should still be managed by social workers or psychologists.

As consistently reported by previous studies, privacy and lack of time in the pharmacy are important barriers [10,11,15,17]. Even though every Belgian pharmacy is required to have a separate room, we know from practice that it is not always used for confidential conversations, or that pharmacists find it difficult to invite patients to have a conversation in such a separate room. Time and privacy issues, which are also related to adequate staffing, are general problems in pharmacy and deserve further in-depth exploration on what future long-term directions are needed for this.

From previous studies, lack of knowledge about the topic appeared to be a factor which hindered pharmacists in providing primary mental health services [11,16,17,18,19]. This was not a barrier in the current study as pharmacists received mandatory online training in psychosocial health care, focusing on common psychosocial problems, the available care offer, and specific conversation techniques. A brief satisfaction survey showed that all pharmacists were rather satisfied to very satisfied with the e-learning content.

It should be noted that community pharmacists enrolled themselves for participation and a selection bias may therefore be present. Thus, the participants may have an affinity for psychosocial wellbeing, and, as a result, the sample may be an exceptionally motivated group. No demographic information was collected about the participating pharmacists, nor information about their work experience, so it is unclear how representative our sample of pharmacists is. Finally, the pharmacies were located in different Flemish regions, but no pharmacy was located in a large urban city. It is likely that the level or distribution of psychosocial needs would be higher in urban areas, but the data do not allow comparisons between regions. Also, we did not further investigate differences between pharmacists who did or who did not actively engage in the project (e.g., registering no contact or many contacts) in terms of demographics. These research questions, such as the effect of urbanicity, can be better examined in the next project phase with a geographically broader implementation.

Regarding the qualitative research, it should be noted that less than half of the participating pharmacists agreed to participate in a focus group. The focus groups were launched rather soon after the start and took place during the pilot phase, so not all barriers may have become clear by then. Also, the online implementation of the focus groups may have caused interactive dynamics to become blurred.

It would also be interesting to conduct additional qualitative research about the general role of pharmacists in psychosocial care with other pharmacists who did not participate or have a completely different view. It is reasonable to assume that pharmacists who think that a role in psychosocial needs detection is beyond their scope of practice will not participate in such a project, so their perspectives are not included in our qualitative discussions with participating pharmacists only.

The patient’s perspective must be further explored in future research. For example, it is unclear whether citizens are open to having their pharmacist address them about possible psychosocial problems and suggest help. The reasons and predictors for this should be identified. Possibly there are patients who appreciate this, while others prefer to discuss these topics with a general practitioner or an informal support network. One qualitative study with patients participating in a mental illness and addictions program showed that patients highly valued the social support and navigation in the mental health care system by their community pharmacist, and they reported significant contributions of the pharmacists in their overall wellbeing [24].

Practice-oriented training for pharmacists on psychosocial wellbeing and care is crucial. An interactive e-learning was therefore already developed for the further implementation of the project and the possibility to incorporate the theme in the undergraduate training of pharmacists will also be investigated, so that in the long run, every pharmacist is able to help patients with psychosocial needs in an appropriate way. In Belgium, there is still much room for improvement in this regard. Mental health is part of the curriculum, in that sense that pharmacists get courses on the pathophysiology and pharmacotherapy of mental disorders, and on their role in counselling patients on the use of specific medicines in this regard. However, dealing with mental health problems and psychosocial wellbeing, i.e. approaching patients with sings of mental health issues or psychosocial issues, or talking about referral, is less well implemented in the curricula. This also holds internationally. As an example, two thirds of undergraduate pharmacy students in the UK and Ireland felt that their degree does not adequately prepare them to help people with their mental health [25]. Nonetheless, they thought that mental health is an important topic and recognize the potential of the pharmacist, but stigma and a lack of applied knowledge and conversational skills need to be addressed first [26]. ‘Mental Health First Aid’ trainings are especially helpful to improve the competencies of pharmacy students and pharmacists and increase their confidence in helping patients with mental health problems, also when patients experience a crisis or suicidal thoughts [27].

Moreover, further efforts should be made to make the collaboration between local psychosocial workers and pharmacists more sustainable and to promote the personal acquaintance and trust between the professional groups. For example, networking events between pharmacists, psychosocial workers and other local primary care providers can be helpful in this regard.

Overall, these findings closely align with the conclusion of a 2015 article about the management of psychosocial problems in Flanders, as it was stated that there is a large need for more and better collaboration, communication and coordination between the actors involved in health care and welfare, as well as the stimulation of research regarding psychosocial problems in primary care in Flanders [5].

## Lessons learned

- Pharmacists can be valuable partners in the detection and referral of patients and pharmacy visitors with an unmet need for psychosocial care
- The role of pharmacists in the detection and referral of psychosocial needs requires sufficient training of pharmacists in psychosocial wellbeing, and this would ideally be included in the university curriculum
- Policymakers should further explore how to address major barriers in general pharmacy practice such as time, privacy and staffing issues

## Conclusion

Psychosocial needs (i.e., any combination of social, emotional or behavioral problems) often remain unmet. Pharmacists are accessible first-line care providers and are therefore in a good position to detect these needs. This study, which was part of a pilot project in Flanders, showed that community pharmacists can successfully help their patients with psychosocial needs by addressing these problems and referring their patients to professional psychosocial help if needed.

We found that the participating pharmacists were willing and able to implement psychosocial care into their role. During the four month study phase since October 2021, a total of 79 patient contacts concerning psychosocial wellbeing were registered. The majority of the patients were middle-aged and female, family and mental health problems accounted for half of the conversations. Focus group discussions with participating pharmacists showed that they are driven by perceived patient satisfaction, but that time and privacy constraints in the pharmacy are important barriers.

In conclusion, structural collaborations between community pharmacy and psychosocial care are promising and need further exploration. It is an example of a good practice of integrated care, with the health and social care sector working together at a local level and with extra attention to the most vulnerable in society.
